# Feeding Cannabidiol (CBD)-Containing Treats Did Not Affect Canine Daily Voluntary Activity

**DOI:** 10.3389/fvets.2021.645667

**Published:** 2021-04-29

**Authors:** Elizabeth M. Morris, Susanna E. Kitts-Morgan, Dawn M. Spangler, Jessica Gebert, Eric S. Vanzant, Kyle R. McLeod, David L. Harmon

**Affiliations:** ^1^Department of Animal and Food Sciences, University of Kentucky, Lexington, KY, United States; ^2^College of Veterinary Medicine, Lincoln Memorial University, Harrogate, TN, United States

**Keywords:** cannabidiol, canine, behavior, activity, pruritus

## Abstract

Growing public interest in the use of cannabidiol (CBD) for companion animals has amplified the need to elucidate potential impacts. The purpose of this investigation was to determine the influence of CBD on the daily activity of adult dogs. Twenty-four dogs (18.0 ± 3.4 kg, 9 months−4 years old) of various mixed breeds were utilized in a randomized complete block design with treatments targeted at 0 and 2.5 mg (LOW) and at 5.0 mg (HIGH) CBD/kg body weight (BW) per day split between two treats administered after twice-daily exercise (0700–0900 and 1,700–1,900 h). Four hours each day [1,000–1,200 h (a.m.) and 1,330–1,530 h (p.m.)] were designated as times when no people entered the kennels, with 2 h designated as Quiet time and the other 2 h as Music time, when calming music played over speakers. Quiet and Music sessions were randomly allotted to daily a.m. or p.m. times. Activity monitors were fitted to dogs' collars for continuous collection of activity data. Data were collected over a 14-day baseline period to establish the activity patterns and block dogs by activity level (high or low) before randomly assigning dogs within each block to treatments. After 7 days of treatment acclimation, activity data were collected for 14 days. Data were examined for differences using the MIXED procedure in SAS including effects of treatment, day, session (Quiet or Music), time of day (a.m. or p.m.), and accompanying interactions. CBD (LOW and HIGH) did not alter the total daily activity points (*P* = 0.985) or activity duration (*P* = 0.882). CBD tended (*P* = 0.071) to reduce total daily scratching compared with the control. Dogs were more active in p.m. sessions than in a.m. sessions (*P* < 0.001). During the p.m. session, dogs receiving HIGH tended (*P* = 0.091) to be less active than the control (CON). During the a.m. and p.m. sessions, CBD reduced scratching compared with CON (*P* = 0.030). CBD did not affect the activity duration during exercise periods (*P* = 0.143). These results indicate that, when supplemented with up to 4.5 mg CBD/kg BW/day, CBD does not impact the daily activity of adult dogs, but may exert an antipruritic effect.

## Introduction

Pet owners and caretakers are increasingly interested in monitoring their animals' behavior and activity as indicators of health and well-being. While several subjective measures like the Canine Brief Pain Inventory and the Hudson Visual Analog Scale are available for use (1, 2), the ability to measure activity through objective, non-invasive means such as with accelerometers is a potentially preferable tool that can provide an impartial measure of animal activity (3–6). The use of accelerometers, kinesiology, and gait analysis are becoming popular methods by which to evaluate the health status of an animal as well as response to treatment. Several triaxial accelerometers have been validated for the measurement of canine activity and can be easily attached to a collar or harness for home use (7–10). They have been used to evaluate the effectiveness of treatments for osteoarthritis and pruritic behaviors (11–14), the effects of exercise and rest on the voluntary activity of active sled dogs (15, 16), and to predict rest in dogs and sleep in humans (17–19).

Normal activity of healthy dogs is influenced by many factors, including breed, age, degree of socialization, and amount of exercise (20–22). Additionally, canine activity may be negatively influenced by factors such as disease, chronic illnesses like osteoarthritis, or behavioral issues such as anxiety (11, 23, 24). There are also certain circumstances where canine activity needs to be reduced as a result of normal activity in high-energy dogs, pruritic behaviors like scratching, or anxious behaviors like pacing and destruction. Activity may also need to be prevented or reduced following an illness, medical treatment, or surgical procedure (13, 24). In such instances, many turn to medications like sedatives or antidepressants that have been shown to reduce canine activity (25). However, some pet owners may be hesitant to turn to such medications due to potential side effects, cost, or personal bias against their use (26, 27). Instead, they often investigate alternatives to conventional medications, such as cannabidiol (CBD).

Cannabidiol is one of over 100 known cannabinoids produced in the glandular trichomes of *Cannabis sativa*. There has been considerable interest in the use of CBD for both humans and companion animals due to its reported benefits, such as analgesia, anti-inflammatory, anxiolytic, and sedative effects (28–30). The analgesic effect of CBD has been documented in rodent and human models (31–33), and the use of oral and transmucosal CBD oil formulations increased the Canine Brief Pain Inventory (CBPI) and Hudson scores in dogs with osteoarthritis, suggesting an increase in activity and comfort with CBD use (34, 35). However, despite evidence of an anxiolytic effect of CBD in both rodents and humans with doses ranging from 2.5 to 10 mg/kg (36–38), a recent report failed to demonstrate an anxiolytic effect of treats containing 1.4 mg CBD/kg body weight (BW) in dogs exposed to a noise-induced fear response test (39). Other effects attributed to CBD, such as sedative effects, are thought to be biphasic. Larger doses have been shown to exert sedative effects in both rats and humans, whereas low doses of CBD may increase wakefulness (40–42). While the effect of CBD on sedation has not been specifically investigated in a canine model, a preliminary investigation of the safety of escalating CBD doses in 20 healthy dogs reported mild constitutional adverse events recorded for dogs receiving 1.7–64.7 mg/kg CBD oil, which included both lethargy and hyperesthesia (43). A similar investigation into the safety of a 1:20 Δ^9^-tetrahydrocannabinol (THC)/CBD herbal extract reported mild neurological adverse events, like ataxia and delayed hopping, after single and multiple oral doses of 2 and 5 mg/kg CBD extract (44). While adverse events in both studies were mild and rare, they do highlight the potential of CBD to cause undesirable side effects as well as the need for continuing research evaluating the safety and efficacy of CBD use in dogs.

Despite the lack of scientific evidence demonstrating the safety and efficacy of CBD use in dogs, a recent survey of over 1,000 dog owners recruited on social media showed that almost 80% of the owners surveyed had purchased hemp or marijuana products for their dogs to provide pain relief, relieve anxiety, aid with sleep, and treat other health conditions. Many also indicated that they believed hemp products were more effective than conventional medications (26, 27). The study population included owners of both healthy and diseased animals as well as owners that either had or had not ever purchased hemp products for their dogs; however, this is likely an overestimation of the overall hemp use in companion animals due to the surveys being shared primarily within social media groups dedicated to cannabis use in pets. Nevertheless, these surveys provide insight into the overwhelmingly favorable perceptions of pet owners on the safety and efficacy of CBD use in companion animals. Due to this interest in the use of CBD in companion animals, there is a critical need for further evaluation of CBD use in dogs and its potential effects on canine activity. Thus, the objective of the current study was to determine the impact of CBD on the daily activity of healthy adult dogs with the underlying hypothesis that CBD would reduce the overall daily activity of dogs compared with the control. This hypothesis was tested using triaxial accelerometers to measure the activity of dogs receiving two levels of CBD administration compared to a control.

## Materials and Methods

This study was approved by the Lincoln Memorial University (LMU) institutional animal care and use committee (protocol 1911-RES) before the start of the study. All housing and husbandry were provided in accordance with the Animal Welfare Act, the Guide for the Care and Use of Laboratory Animals (8th ed.), and all applicable LMU protocols.

### Subjects and Housing

Thirty neutered adult dogs (15 males, 15 females, 9 months to 4 years old, 17.6 ± 3.4 kg) of various mixed breeds, including terrier, hound, Bassett, shepherd, border collie, husky, cur, lab, boxer, and pug mixes, were received at the LMU DeBusk Veterinary Teaching Center (DVTC) from a local shelter for inclusion in this study. The shelter was asked to provide dogs weighing 16 ± 4 kg. Additionally, the shelter was informed and gave consent for the use of the dogs for research purposes before their arrival. Before beginning the experiment, each dog had a complete blood count (CBC) and serum chemistry analysis (IDEXX Laboratories, Inc., Westbrook, ME) performed, along with a physical evaluation by a veterinarian and a fecal examination to rule out any underlying disease that might preclude enrollment. Dogs were excluded if they demonstrated serious behavioral issues, such as extreme fear or human aggression that would endanger research personnel, were severely emaciated or obese, classified as a body condition score <2 or >4 on a five-point scale (where one is emaciated and five is obese), or if the initial evaluations revealed an underlying disease that required more than routine treatments, such as heartworm infection, metabolic or infectious disease, and mobility issues. Three dogs were excluded due to positive heartworm tests and another three dogs excluded for behavioral concerns. The remaining 24 dogs (12 males, 12 females, 9 months to 4 years old, 18.0 ± 3.4 kg) were selected for inclusion in the study. The dogs were individually housed in 1.2 × 1.8-m cages within one of two dog kennels at the LMU DVTC for the duration of the study.

### Diets and Treatments

Dogs were fed Purina Pro Plan EN Gastroenteric Fiber Balance Dry Dog Food (Nestle Purina Inc., St. Louis, MO) to meet the daily metabolizable energy requirements of neutered adult dogs at maintenance, calculated as (70 × BW^0.75^) × 1.6, and split into two meals per day fed between 0700 and 0900 h and between 1,700 and 1,900 h each day. Dogs were weighed and body condition scored (five-point scale) weekly and the diets adjusted accordingly. Treatments were arranged in a randomized complete block design and consisted of 0 (placebo treats, CON), 34.0 ± 1.16 (LOW), or 75.6 ± 5.86 (HIGH) mg CBD/day. CBD is a constituent of a proprietary industrial hemp extract (AgTech Scientific, Paris, KY) that was incorporated into treats and administered in the form of two treats daily, each containing half the daily dose. Both control and CBD treats were composed of the following ingredients: chicken, chicken liver, Asian carp, catfish, and—in the case of CBD treats—industrial hemp extract. While CBD was the primary constituent of the industrial hemp extract, trace THC was present in both LOW and HIGH treatments (1.1 ± 0.37 and 2.9 ± 0.22 mg THC/day, respectively). Treats were formulated to target CBD at doses of 2.5 and 5.0 mg/kg BW/day for LOW and HIGH treatments, respectively, based on an estimation that dogs would weigh an average of 16 kg. The LOW dose was selected based on previous literature that utilized a similar dose in dogs to assess single-dose pharmacokinetics of CBD and to evaluate its potential to alleviate pain in dogs with osteoarthritis (35). That dose was then doubled to achieve the HIGH dosage. However, based on the mean BW of the dogs included in the study and analysis of the treats, the mean doses of CBD were 1.8 and 4.5 mg CBD/kg BW/day for the LOW and HIGH treatments, respectively. Treats were offered solely as a reward upon kennel reentry following twice-daily exercise, which occurred within 30 min of meals.

### Experimental Design and Data Collection

Upon completion of intake exams, the dogs underwent a 7-day acclimation period for adjustment to the environment, diet, collars, and daily routine (Table 1). Kennels were maintained on a 12-h light schedule. Dogs received two 15-min exercise periods each day, with the morning exercise occurring between 0700 and 0900 h and the evening exercise occurring between 1,700 and 1,900 h. During the exercise periods, dogs that were aggressive toward other dogs were individually hand-walked by research personnel; all other dogs were allowed to exercise freely in playgroups of two to four dogs in one of two adjacent grassy enclosures. The numbers of dogs being hand-walked and those in playgroups were balanced across all treatments. Four hours each day—from 1,000 to 1,200 h (a.m.) and from 1,330 to 1,530 h (p.m.)—were designated as times when no people were allowed to enter the kennels. Two of those 4 h were designated as Quiet time and the other 2 h as Music time, when calming music was played over speakers in each kennel. Quiet and Music sessions were randomly allotted to either a.m. or p.m. time each day. All dogs started receiving control treats (0 mg CBD) twice daily as a reward for kennel reentry after the twice-daily exercise.

**Table 1:** Schedule of events for monitoring activity in dogs receiving cannabidiol-containing treats.

**Day of study**	**Event**	**Data collection**	**Treats**
−2 and −1	Intake and initial health exams	None	Control
1 to 7	Acclimation	None	Control
8 to 21	Baseline period activity collection	Vetrax activated	Control
22 to 28	Treatment adaptation	None	Treatment
29 to 43	Treatment period activity collection	Vetrax activated	Treatment

After the acclimation period, Vetrax® activity sensors (AgLogica Holdings, Norcross, GA) were fitted to dogs' collars using the attachment provided by the manufacturer and placed ventral to the mandible. These triaxial accelerometers were used for the continuous collection of activity variables—activity points, activity duration (in minutes), duration of no activity (in hours), duration of resting (in hours), running duration (in minutes), walking duration (in minutes), scratching duration (in seconds), head shaking duration (in seconds), and sleep quality (Table 2). Data collected by the sensors were automatically uploaded to the Vetrax® server *via* Wi-Fi once an hour for behavior algorithm processing, which has been previously validated (8). Except for a weekly consistent 2- to 3-h charging period, sensors remained on the dogs at all times. Before the start of the experiment, the data were collected over a 14-day baseline period to block dogs by mean daily activity—high (mean = 118.6 min, range = 88.6–157.5 min) or low (mean = 59.3 min, range = 30.2–85.1 min)—before stratifying dogs by age, weight, and sex and randomly assigning dogs within each block to treatments. Dogs were stratified by treatment and sex, evenly distributed between the two kennels, and adapted to treatments for 7 days before another 14-day collection of activity *via* Vetrax® sensors (Table 1).

**Table 2:** Activity variables measured by Vetrax® activity sensors (AgLogica Holdings, Inc., Norcross, GA).

**Variable**	**Definition**
Activity points	Total activity of dogs weighted by each individual activity (e.g., running worth more points than walking), calculated using a proprietary algorithm
Activity (min)	Duration of total activity including running and walking
No activity (h)	Duration of complete inactivity
Resting (h)	Duration of time not actively walking or running, but not completely inactive
Running (min)	Duration of running
Walking (min)	Duration of walking
Scratching (s)	Time spent scratching
Head shaking (s)	Time spent shaking head
Sleep	Scale of sleep quality measured using a proprietary algorithm based on absence of nighttime disturbance; scaled 0 – 100, with 100 being undisturbed sleep

Consumption of food and treats, consistency of stool, frequency of elimination, subjective assessment of activity during exercise, mucus membrane color, and other indicators of general health status were monitored twice daily by research personnel. Evidence of any adverse event—defined as any symptom occurrence that would not be expected in normal dogs—was also monitored. However, no adverse events were observed in any dogs following the administration of CBD treats during this study.

### Statistical Analysis

Based on variations in activity and behaviors reported in previous work using these sensors (8, 13), it was calculated that *n* = 8 dogs/treatment was sufficient to detect a 25% change with a 16% coefficient of variation (CV) (45). Activity monitors for two of the dogs in the control group (one in the high-activity block and one in the low-activity block) spontaneously stopped transmitting halfway through the treatment period, and activity data from the last 7 days of the experiment for those two dogs were lost.

The normality of the residuals was tested using the UNIVARIATE procedure in SAS (SAS Institute, Cary, NC). In instances where the data did not meet normality assumptions, statistical analysis was performed on transformed data. However, the data were then back-transformed for reporting purposes. The standard errors of the back-transformed data were calculated from the confidence limits of the transformed data as follows: SEM = (back-transformed upper limit – back-transformed lower limit)/3.92. The denominator relates to the *Z*-value of a 95% confidence interval (±1.96). For the baseline period, activity duration, running, scratching, and head shaking were not normally distributed and were log-transformed for statistical analysis. Activity points and walking were not normally distributed and were transformed into the square root for statistical analysis.

During the baseline period, dogs allotted to CBD treatments tended (*P* = 0.061) to run more than the control; thus, the mean duration of running from the baseline period was utilized as a covariate for the duration of running in the treatment period. All other variables were similar across treatments in the baseline period. For overall daily activity during the treatment period, running, scratching, and head shaking were not normally distributed and were log-transformed for statistical analysis. For Quiet and Music session activity periods, all variables, except for No Activity and Resting, were not normally distributed and were log-transformed for statistical analysis. For exercise activity periods, activity points, activity duration, and scratching were not normally distributed and were log-transformed for statistical analysis, whereas running and head shaking were not normally distributed and were transformed to the cube root for statistical analysis.

From the treatment period, overall daily activity and activity during the exercise periods (0700–0900 and 1,700–1,900 h) were analyzed using the MIXED procedure in SAS including the fixed effects of treatment, day, and the treatment by day interaction. Dog nested within the activity block (high or low) was included as a random effect and day was included as a repeated measure with dog nested within treatment as the subject. Activity during the Quiet and Music sessions was analyzed using the MIXED procedure in SAS including the fixed effects of treatment, day, session (Quiet or Music), time of day (a.m. or p.m.), and all accompanying interactions. Dog nested within the activity block was again included as a random effect. Time (a.m. or p.m.) was included as a repeated measure with dog nested within treatment as the subject. Treatment effects are described as the contrast between CON and both CBD treatments and the contrast between LOW and HIGH CBD treatments. The results are presented as the mean ± SE. Effects were considered significant when *P* ≤ 0.05 and considered a tendency when *P* < 0.10.

## Results

### Total Daily Activity

CBD did not alter the total activity points, activity duration, no activity, resting, running, walking, head shaking, or sleep quality compared to CON (*P* > 0.05; Table 3). However, CBD tended to reduce scratching compared with CON (*P* = 0.071), but was not different between the LOW and HIGH treatments (*P* = 0.209). The level of CBD inclusion (LOW vs. HIGH) did not affect any variables measured (*P* > 0.05). With the exceptions of activity duration, running, walking, and scratching, all variables were affected by day of treatment (*P* < 0.05), but there were no treatment by day interactions (*P* > 0.05).

**Table 3:** Effect of treatment (TRT), day, and TRT^*^day interaction on total daily activity variables collected *via* Vetrax® activity sensors (AgLogica Technology, Norcross, GA).

	**Treatment**			**SE^b^**	***P*****-value**			
**Variable^a^**	**Control (CON)**	**1.8 mg CBD/kg BW/day (LOW)**	**4.5 mg CBD/kg BW/day (HIGH)**		**CON vs. CBD**	**LOW vs. HIGH**	**Day**	TRT*Day
Activity points	56,834	57,079	56,634	4,952.5	0.985	0.950	0.017	0.857
Activity (min)	80.1	82.7	82.0	11.90	0.882	0.966	0.421	0.773
No activity (h)	13.6	13.9	14.3	0.58	0.496	0.652	0.002	0.394
Resting (h)	8.8	8.5	8.1	0.43	0.364	0.513	<0.001	0.389
Running (min)	5.8	5.6	6.9	0.36	0.612	0.172	0.172	0.447
Walking (min)	76.2	76.5	71.8	10.94	0.878	0.760	0.531	0.749
Scratching (s)	69.6	35.8	51.9	7.33	0.071	0.209	0.162	0.485
Head shaking (s)	32.4	27.3	42.4	5.49	0.878	0.229	0.006	0.194
Sleep quality	77.2	76.1	75.1	2.21	0.500	0.722	0.029	0.679

*Treatment effects are shown as the contrast between control (CON) and both CBD treatments (CBD) and the contrast between CBD treatments (LOW vs. HIGH)*.

a *Running, scratching, and head shaking were not normally distributed and were transformed for statistical analysis. Data were back-transformed for reporting purposes*.

b *Standard error (SE) of the back-transformed data was calculated as follows: SE = (back-transformed upper limit – back-transformed lower limit)/3.92*.

### Quiet and Music Session Activity

Overall, dogs were more active in the p.m. sessions than in the a.m., with all variables affected by the time of day (*P* < 0.001; Table 4). Activity points, activity duration, running, walking, and resting increased in the p.m. compared to the a.m. (*P* < 0.001), while the duration of No Activity decreased in the p.m. compared to a.m. (*P* < 0.001). During these sessions, the Music session tended to reduce activity points (*P* = 0.055) and running (*P* = 0.098) compared to the Quiet session. The Music session reduced activity duration (*P* = 0.002), walking (*P* < 0.001), and resting (*P* = 0.045) while increasing the duration of no activity (*P* = 0.014) compared to the Quiet session.

**Table 4:** Effect of treatment (TRT), day, session (Quiet or Music), time of day (a.m. or p.m.), and all relevant interactions on activity variables collected *via* Vetrax® activity sensors (AgLogica Technology, Norcross, GA) at 1,000–1,200 h (a.m.) and 1,330–1,530 h (p.m.) each day.

	**Treatment**				***P*****-value**							
**Variable^a^**	**Control (CON)**	**1.8 mg CBD/kg BW/day (LOW)**	**4.5 mg CBD/kg BW/day (HIGH)**	**SE^b^**	**CON vs. CBD**	**LOW vs. HIGH**	**Time of day**	**Session**	Session*time	TRT*time	TRT*session	**Day**
Activity points	2,884	2,509	2,308	148.6	0.152	0.550	<0.001	0.055	0.014	0.287	0.465	<0.001
Activity (min)	1.23	0.87	0.85	0.144	0.204	0.937	<0.001	0.002	0.076	0.079	0.257	<0.001
No activity (h)	1.21	1.25	1.34	0.059	0.273	0.327	<0.001	0.014	<0.001	0.013	0.443	0.002
Resting (h)	0.71	0.70	0.62	0.050	0.384	0.234	<0.001	0.045	<0.001	0.006	0.203	0.002
Running (min)	0.04	0.03	0.04	0.006	0.602	0.544	<0.001	0.098	0.078	0.369	0.834	0.223
Walking (min)	1.29	0.87	0.82	0.148	0.138	0.857	<0.001	<0.001	0.084	0.060	0.217	<0.001
Scratching (s)	4.18	2.29	2.62	0.413	0.030	0.612	<0.001	0.930	0.274	0.326	0.886	0.730
Head shaking (s)	1.78	1.65	1.82	0.128	0.882	0.600	<0.001	0.926	0.305	0.319	0.212	0.375

*Treatment effects are shown as the contrast between control (CON) and both CBD treatments (CBD) and the contrast between CBD treatments (LOW vs. HIGH)*.

a *Except for No Activity and Resting, variables were not normally distributed and were transformed for statistical analysis. Data were back-transformed for reporting purposes. There was no effect of the treatment by day or the session by time by treatment interactions on any variable (P > 0.05) and thus are not shown*.

b *Standard error (SE) of the back-transformed data was calculated as follows: SE = (back-transformed upper limit – back-transformed lower limit)/3.92*.

Session by time interactions were observed for activity points, no activity, and resting (*P* < 0.05; Table 4), and a trend for session by time interactions was observed for activity duration, running, and walking (*P* = 0.076, 0.078, and 0.084, respectively). The type of session (Quiet or Music) did not alter activity points or duration of activity during the a.m. session (*P* = 0.502 and 0.522, respectively). When the Quiet session was allotted to the p.m., however, activity points (*P* = 0.002), duration of activity (*P* < 0.001), resting (*P* < 0.001), running (*P* < 0.001), and walking (*P* < 0.001) were increased compared to when the Music session was allotted to the p.m. The durations of No Activity were similar between the Quiet and Music sessions when allotted to the a.m. (*P* = 0.230), but the duration was increased in the Music session compared to the Quiet session when allotted to the p.m. (*P* < 0.001).

Activity points, running, and head shaking were unaffected by treatment and all treatment interactions during the Quiet and Music sessions (*P* > 0.05; Table 4). Scratching was reduced by CBD during the Quiet and Music sessions compared to CON (*P* = 0.030), but the level of CBD inclusion did not affect time spent scratching (*P* = 0.612).

A treatment by time interaction was observed for No Activity and Resting (*P* = 0.013 and 0.006, respectively; Table 4), and a trend for a treatment by time interaction was observed for activity and walking duration (*P* = 0.079 and 0.060, respectively). Regardless of Quiet or Music session, activity durations were similar across treatments in the a.m. (*P* > 0.05), but dogs receiving HIGH CBD tended (1.44 ± 0.172 min, *P* = 0.091) to be less active than CON (2.64 ± 0.324 min) in the p.m. and tended (1.37 ± 0.162 min, *P* = 0.059) to walk less than CON (2.60 ± 0.326 min) in the p.m. Similarly, the duration of No Activity was unaffected by treatment in the a.m. (*P* > 0.05), but in the p.m. tended to increase in the HIGH CBD treatment (1.13 ± 0.060 h) compared to both CON (0.95 ± 0.063 h) and LOW (0.97 ± 0.060 h) treatments (*P* = 0.054 and 0.068, respectively). Conversely, resting duration increased in the LOW treatment (0.96 ± 0.051 h) compared to the HIGH treatment (0.80 ± 0.051 h) in the p.m. (*P* = 0.038), but similar across all other time points and treatments (*P* > 0.05). There were no treatment by session nor treatment by session by time interactions (*P* > 0.05) for any variables measured. All activity variables were affected by day of treatment period (*P* < 0.05), but there were no treatment by day interactions (*P* > 0.05).

### Exercise Activity

Neither CBD treatment nor inclusion level affected any variables measured during the exercise periods (*P* > 0.05; Table 5). Day of treatment period tended (*P* = 0.066) to affect scratching and affected head shaking (*P* = 0.003), but no other variables were impacted by day of treatment (*P* > 0.05). Additionally, there were no treatment by day interactions (*P* > 0.05).

**Table 5:** Effect of treatment (TRT), day, and TRT^*^day interactions on activity parameters collected *via* Vetrax® activity sensors (AgLogica Technology, Norcross, GA) during the two periods of daily exercise, which included all data from 0700–0900 to 1,700–1,900 h each day.

	**Treatment**				***P*****-value**			
**Variable^a^**	**Control (CON)**	**1.8 mg/kg BW/day CBD (LOW)**	**4.5 mg/kg BW/day CBD (HIGH)**	**SE^b^**	**CON vs. CBD**	**LOW vs. HIGH**	**Day**	TRT*day
Activity points	21,736	25,735	26,122	2,096.8	0.143	0.910	0.117	0.283
Activity (min)	37.66	48.09	48.07	3.213	0.143	0.998	0.528	0.305
No activity (h)	0.82	0.75	0.77	0.132	0.708	0.899	0.359	0.842
Resting (h)	2.47	2.24	2.31	0.116	0.312	0.838	0.338	0.847
Running (min)	4.9	4.5	5.3	0.31	0.940	0.298	0.258	0.531
Walking (min)	35.0	43.8	40.7	0.12	0.207	0.446	0.958	0.576
Scratching (s)	21.1	14.1	20.3	2.53	0.442	0.267	0.066	0.875
Head shaking (s)	17.8	14.7	25.8	0.27	0.682	0.167	0.003	0.273

*Treatment effects are shown as the contrast between control (CON) and both CBD treatments (CBD) and the contrast between CBD treatments (LOW vs. HIGH)*.

a *Except for No Activity and Resting, variables were not normally distributed and were transformed for statistical analysis. Data were back-transformed for reporting purposes*.

b *Standard error (SE) of the back-transformed data was calculated as follows: SE = (back-transformed upper limit – back-transformed lower limit)/3.92*.

## Discussion

Triaxial accelerometer sensors were used in this study to determine the effect of daily CBD dosing on activity in healthy adult dogs by measuring daily activity, pruritic behaviors, and an assessment of rest and sleep quality. The objective of this study was to evaluate the impact of CBD on the daily activity of healthy adult dogs with the hypothesis that CBD would reduce the overall daily activity compared to the control. However, the results showed that oral CBD administration did not alter the overall daily activity of healthy adult dogs. The lack of effect on overall daily activity and sleep quality was unexpected based on previous reports of the sedative and hypnogenic effects of CBD in rodent, human, and canine models. In humans and rats, CBD doses ranging from ~2 to 40 mg/kg BW/day have been reported to induce sedative effects, improve sleep quality, and increase total sleep time (41, 46, 47). However, more recent work has reported CBD to have no influence on the sleep cycle in humans (48), and others argue that CBD by itself does not produce sedative effects but rather modulates the sedative effect of Δ^9^-tetrahydrocannabinol (THC), even if THC is only present in minute amounts (42, 49, 50).

The potential for the sedative effect of CBD to be caused by the presence of THC may be supported by an escalating dose study in dogs where placebo, CBD-predominant, THC-predominant, and CBD/THC combination oils were administered to dogs to evaluate the occurrence and severity of adverse events after administration (43). Doses for the CBD-predominant oil started at 1.7 mg CBD/kg BW/day and were incrementally increased to a maximum of 64.7 mg CBD/kg BW/day over 30 days. Lethargy was reported with the CBD-predominant oil formulation. However, that oil was not THC-free; it was reported to contain 0.7 mg/ml THC (43). The industrial hemp extract included in the CBD treats used in this experiment contained a similar THC content to the oil reported in Vaughn et al. (43), but did not produce a similar effect. The reason for these conflicting results remains unclear. These differences could be due to the difference in animals utilized for the study—shelter vs. research-bred dogs—or the different modes of delivery—eating a treat *vs*. oral gavage of an oil. There have been reports of variations in the pharmacokinetics of CBD depending on the mode of delivery. In one experiment, CBD-infused oil demonstrated an increased maximum plasma CBD concentration compared to the same dose administered as microencapsulated oil beads and a CBD-infused transdermal cream (51). Other reports using similar doses of oral CBD oil and chews showed an increased maximum plasma CBD concentration when administered as a chew compared to an oil; however, this has yet to be investigated in a single, controlled experiment (35, 52). Additionally, the dogs used in Vaughn et al. (43) fasted before the administration of CBD oil, whereas the dogs in the current experiment consumed CBD treats within 30 min of a meal. It has been suggested that administering cannabinoids with a fat meal increased bioavailability (53). Since the CBD used in Vaughn et al. (43) was mixed in a lipid-based formulation, it is unclear whether these differences in methodology would lead to the difference in the sedative effects observed between their report and the current study. Additional investigation using THC-free CBD is needed to evaluate the potential for CBD to exert a sedative effect in dogs.

While there was no observed effect on the overall daily activity with CBD treatment, it tended to influence activity during different times of the day. The dogs in the current study were more active in the p.m. than in the a.m. regardless of treatment and type of session. Playing calming music in the kennels (Music session) did reduce activity compared to when no music was played (Quiet session), which supports previous work showing that playing music can reduce stress and increase relaxed behaviors in kenneled dogs (54–56). This effect, however, appears to be independent of the effect of CBD as there was no interaction between treatment and session nor a treatment by session by time interaction. The tendency for dogs in the HIGH CBD treatment to be less active than CON dogs in the p.m. may indicate that CBD exerted some sedative or calming effect on the dogs. However, this potential sedative effect was expected to be observed in the a.m., as previous pharmacokinetic reports have shown a half-life for CBD of 1–4 h (35, 51, 52, 57). As this effect was not observed during the a.m. sessions, exercise periods, or overall daily activity, these collective results do not support a sedative or calming effect of CBD in dogs. Thus, the claim that CBD exerts a sedative or calming effect in dogs remains unsubstantiated, but further investigation may provide clarification of these results.

In the present study, dogs were necessarily regimented into a strict schedule of daily activities. It is possible that, in a setting where dogs were entirely free to choose their activities, such as a home, the outcome could have been different. The strict, consistent schedule of the kennel environment did not allow for much activity outside of the scheduled exercise periods, which may have prevented normally high-energy dogs from being as active as they could be with consistent free access to more space. Conversely, shelter environments have been shown to increase activity in dogs compared to a home environment and may prevent dogs from resting due to increased stress (58, 59). This may have artificially increased activity in dogs that would have otherwise been less active. As a result, it may be preferable to evaluate the effect of these treatments in familiar environments that have more space for dogs to exhibit normal activity and rest behaviors. Additionally, the small sample size and the use of healthy adult dogs were limitations of this study. The dogs included in this study exhibited high variability in voluntary activity despite being blocked by baseline activity and having no known mobility or behavioral issues. These limitations may preclude the extrapolation of these results to other canine populations. Since CBD is often used to increase comfort and activity in dogs with mobility issues like osteoarthritis or to decrease the activity of anxious or hyperactive dogs (27), future work should evaluate voluntary activity in animals with mobility or behavioral issues like osteoarthritis or anxiety.

The results from this study suggest a potential antipruritic effect of CBD. Phytocannabinoids like CBD act on the body through the endocannabinoid system (ECS), which is a signaling system including endocannabinoids like anandamide and 2-arachidonylglycerol, their receptors, and regulatory enzymes (60). The ECS helps regulate metabolic homeostasis, thermoregulation, epidermal homeostasis, and more (61, 62). While CBD has little to no affinity for CB1 and CB2 ECS receptors, it is a known agonist for the transient receptor potential vanilloid family of receptors (TRPV1-4), which are known ECS receptors widely expressed in the skin and play a role in itch sensation (61, 63–65). As TRPV1 is rapidly desensitized after activation, it is thought that CBD may exert antipruritic effects by keeping TRPV1 desensitized, thus preventing neuronal activation by irritants (66–68). Additionally, CBD has been shown to be an antagonist for transient receptor potential melastatin 8 (TRPM8) receptors (67, 69). In the skin, TRPM8 is responsible for environmental cold detection and has been suggested to contribute to the perception of pain and itch, which may indicate that it is another target for the potential antipruritic effect of CBD (64, 70). The antipruritic effect of cannabinoids has been observed in humans (71–73), but this is the first report of a potential antipruritic effect of CBD in dogs as a reduction in scratching duration was observed in dogs. While this experiment was not designed to assess the antipruritic effect of CBD, these results may suggest a potential for CBD to be beneficial in the treatment of skin conditions and pruritic behaviors in dogs. To investigate this potential effect, it would be beneficial for future work to specifically examine the effect of CBD in dogs with skin issues such as allergies, atopic dermatitis, or unexplained pruritus.

## Conclusions

The results of the current study indicate that when supplemented with up to 4.5 mg/kg BW/day, CBD does not impact the overall daily activity of adult dogs. Total daily activity including duration of the activity, sleep quality, and resting were unaffected by CBD. Similarly, activity during the exercise periods was also unaffected by CBD. During the Quiet and Music session periods, 4.5 mg CBD/kg BW/day tended to reduce activity in dogs compared to both 1.8 mg CBD/kg BW/day and CON, but this did not translate to an overall daily effect. Playing classical music in the kennels reduced activity compared to having no music played, but did not alter the response to CBD. CBD reduced total daily scratching as well as scratching during the Quiet and Music sessions, which may indicate a possible antipruritic effect. Future work examining the effect of CBD on activity is warranted, particularly in dogs with mobility and behavioral issues like osteoarthritis and anxiety. Additionally, the potential antipruritic effect of CBD should be investigated using dogs with dermatological issues like skin allergies or atopic dermatitis.

## Data Availability Statement

The raw data supporting the conclusions of this article will be made available by the authors, without undue reservation.

## Ethics Statement

The animal study was reviewed and approved by Lincoln Memorial University institutional animal care and use committee (protocol 1911-RES). Written informed consent was obtained from the owners for the participation of their animals in this study.

## Author Contributions

DH, EM, KM, and SK-M contributed to the conception and design of the study. EM, SK-M, DS, and JG facilitated data collection. EV and EM performed statistical analysis. EM wrote the first draft of the manuscript. All authors contributed to manuscript revision, read, and approved the submitted version.

## Conflict of Interest

The authors declare that the research was conducted in the absence of any commercial or financial relationships that could be construed as a potential conflict of interest.
